# Exosomal PD-L1 and lactate versus tissue PD-L1 as biomarkers for clinical outcomes of PD-1 Blockade plus chemotherapy in metastatic esophagogastric signet ring cell carcinoma

**DOI:** 10.1186/s40164-025-00615-w

**Published:** 2025-03-12

**Authors:** Yuanyuan Tian, Wei Shi, Jing Wang, Wenjie Zhang, Lingling Xia, Lijuan Gao, Hu Qiu, Zhenhua Yu, Yongfeng Zhang, Yongshun Chen

**Affiliations:** 1 Cancer Center, Renmin Hospital of Wuhan University, Wuhan, China; 2 Department of Oncology, The First Affiliated Hospital of Henan University, No. 357, West Gate Street, Kaifeng, 475000 China; 3 Cancer Center, The Eighth Affiliated Hospital, Sun Yat-sen University, No. 3025, Shennan Middle Road, Shenzhen, 518033 China; 4 Pathology Department, The First Affiliated Hospital of Henan University of Science and Technology, 24, Jinghua Road, 471000 Luoyang, China; 5 School of Physics Science and Technology, Wuhan University, Wuhan, China

## Abstract

In this investigator-initiated, prospective, exploratory study, biomarkers predictive of clinical outcomes of first-line immune checkpoint inhibitor (ICI, nivolumab or pembrolizumab) plus XELOX(oxaliplatin and capecitabine) were identified in human epidermal growth factor receptor 2 (HER2)–negative patients with metastatic esophagogastric signet ring cell carcinoma. The findings showed an objective response rate (ORR) of 51.5% and a disease control rate of 86.8%, the median progression-free survival (PFS) for the entire cohort was 6.63 months. PD-L1 expression level in tumor tissues could not identify a high PD-L1 group that significantly benefited from ICI plus XELOX in terms of the ORR and PFS. By contrast, the patients expressing low exosomal PD-L1 or lactate in peripheral blood plasma before treatment initiation demonstrated a significantly increased ORR and prolonged PFS compared to that with high exosomal PD-L1 or lactate, patients with combining predictor of exosomal PD-L1 and lactate lower than − 0.249 was associated with a better ORR (82.1% vs. 30.0%, *P* < 0.001) and a longer median PFS (13.83 vs. 5.50 months, *P* < 0.001) compared to those with combining predictor ≥–0.249. The results also revealed that exosomal PD-L1 levels in peripheral blood plasma before the treatment were significantly correlated with the frequency of CD8^+^ T cells (*P* = 0.007), and in patients after receiving ICI plus XELOX, high exosomal PD-L1 level was associated with more PD-1^+^ Treg cells, high exosomal lactate level was associated with less CD8^+^ T cells and more Treg cells. Thus, the levels of PD-L1 and lactate in exosomes may affect the balance between Treg cells and CD8^+^T cells, leading to treatment resistance to ICI plus XELOX. Compared to PD-L1 expression level in tumor tissues, exosomal PD-L1 and lactate levels could more accurately predict clinical outcomes of HER2–negative patients with metastatic esophagogastric signet ring cell carcinoma receiving first-line PD-1 blockade plus chemotherapy.

**Supplementary Information:**

The online version contains supplementary material available at 10.1186/s40164-025-00615-w.

To the editor,

Signet ring cell carcinoma (SRCC) is a specific type accounting for 8–30% of esophagogastric cancer patients, and advanced SRCC has a worse prognosis [1, 2]. PD-1 antibodies have shown encouraging anti-tumor activity and acceptable safety in all types of esophagogastric cancer [3–5]. Given the lack of prospective data evaluating predictive factors that are associated with the therapeutic efficacy of anti-PD-1 on SRCC, we aimed to compare exosomal PD-L1 and lactate versus tissue PD-L1 and assess if either provided utility as a predictive biomarker in HER2–negative patients with metastatic esophagogastric SRCC receiving first-line PD-1 blockade plus chemotherapy. Additionally, we sought to explore the correlation between PD-L1 and lactate levels in exosomes and peripheral blood T cells subsets.

From January 2022 to March 2023, 68 HER2–negative patients with metastatic esophagogastric SRCC were included, the median age was 63 years old, 67.6% patients were male, ECOG PS of 1 score (72.1%), HER2(0) (57.4%), microsatellite instability status (stable) (98.5%) and metastatic disease of the stomach (66.2%). As of the cut-off date (June 1, 2024), the median follow-up time was 14.2 months. The efficacy noted an objective response rate (ORR: CR + PR) of 51.5% and a disease control rate of 86.8% (Fig. 1, supplementary Tables 1, 2). Materials and methods of this study can be found in the supplementary data.

**Fig. 1:**
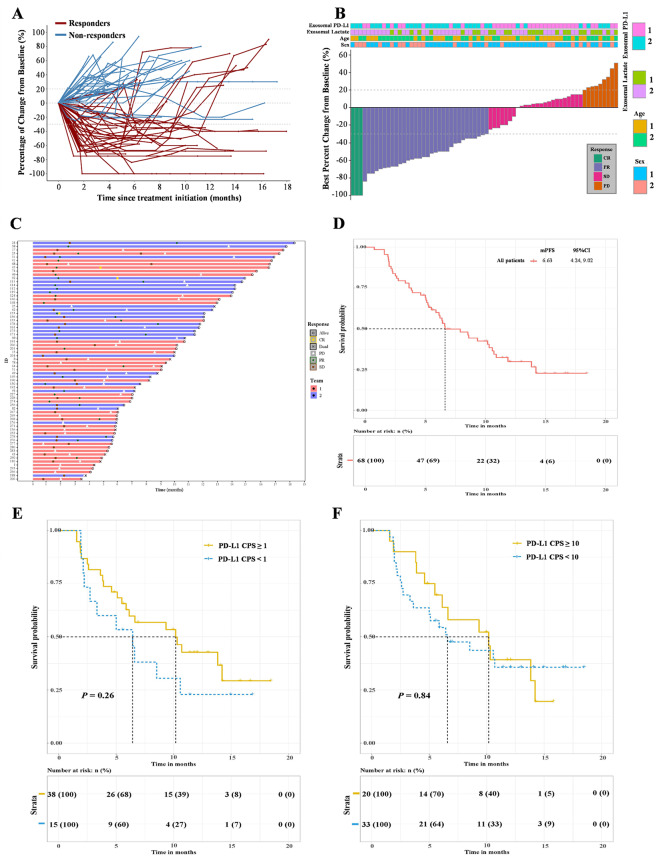
Treatment efficacy. (**A**) Spider plot of tumor response in target lesions of 68 patients. (**B**) Waterfall plots showing the best change of target lesion from baseline. *Note*: Exosomal PD-L1, 1: high exosomal PD-L1 group (exosomal PD-L1 ≥ 55.237 pg/ml), 2: low exosomal PD-L1 group (exosomal PD-L1 < 55.237 pg/ml); Exosomal lactate, 1: high exosomal lactate group (exosomal lactate ≥ 3.681 ng/ml), 2: low exosomal lactate group (exosomal lactate < 3.681 ng/ml); Age, 1: age < 65 years, 2: age ≥ 65 years; Sex, 1: male, 2: female. (**C**) Swimming chart showing the treatment results of the low combination prediction group (*n* = 28) and the high combination prediction group (*n* = 40). *Note*: 1: combining predictor of exosomal PD-L1 and lactate ≥–0.249, 2: combining predictor of exosomal PD-L1 and lactate <–0.249. (**D**) Kaplan-Meier estimates of progression-free survival in the entire cohort. (**E**, **F**) Correlations between tumor PD-L1 CPS and progression-free survival. *P*-values were based on a two-sided log rank test for survival analysis

The levels of exosomal PD-L1 or lactate in plasma before treatment initiation in responders (CR + PR) were much lower than that in non-responders (SD + PD) (*P* < 0.05), while no significant correlation was observed between tissue PD-L1 CPS and tumor response (*P* = 0.103) (supplementary Fig. 1). Exosomal PD-L1 < 55.237 pg/ml or exosomal lactate < 3.681 ng/ug before treatment associated with a better response to first-line PD-1 blockade plus XELOX by ORR (*P* < 0.01), the combining predictor of exosomal PD-L1 and lactate lower than − 0.249 was associated with a better response to the treatment by ORR (82.1% vs. 30.0%, *P* < 0.001). The median PFS for the entire cohort was 6.63 months, patients with a PD-L1 CPS ≥ 1 or ≥ 10 achieved a longer PFS compared to those with PD-L1 CPS < 1 or < 10, but there was not statistically different. However, compared to those with high level of exosomal PD-L1 or exosomal lactate, patients presented with exosomal PD-L1 < 55.237 pg/ml or exosomal lactate < 3.681 ng/ug before treatment achieved a prolonged mPFS (*P* < 0.01), patients with the combining predictor of exosomal PD-L1 and lactate lower than − 0.249 achieved a much longer mPFS when compared to that with high level of the combining predictor (13.83 vs. 5.50 months, *P* < 0.001) (Fig. 2). The same trend was observed in 92 patients used for external validation (supplementary Fig. 2). We investigated whether T cells and their co-expressed PD-1 in the blood can predict response to treatment, and found that the frequency of CD8^+^ T cells of the responders was significantly higher than that of the non-responders (*P* = 0.002), while the frequency of CD4^+^ T cells (*P* = 0.001), Treg cells (*P* = 0.002), PD-1^+^ CD8^+^ cells (*P* = 0.030) and PD-1^+^ Treg cells (*P* < 0.001) of the responders were significantly lower than that of the non-responders. After treatment, the ratio of CD8^+^ T cells significantly increased in responders (*P* = 0.049), while the ratio of Treg cells significantly increased in non responders (*P* = 0.049) (supplementary Fig. 3). Exosomal PD-L1 level was negatively correlated with the frequency of CD8^+^T cells (*P* = 0.007) and positively correlated with the frequency of CD4^+^T cells (*P* = 0.008) and the rate of CD4^+^T cells/CD8^+^T cells (*P* = 0.010), exosomal lactate level was positively correlated with the rate of Treg cells/CD8^+^T cells (*P* = 0.036). Correlation analysis shows that high exosomal PD-L1 level was associated with more PD-1^+^ Treg cells (*P* = 0.005) and more Treg cells (*P* = 0.041), while high exosomal lactate level was significantly associated with an increase in the ratio of Treg cells/CD8^+^ T cells (*P* = 0.034), associated with more Treg cells and fewer CD8^+^T cells after treatment, the combination of exosomal PD-L1 and lactate best distinguished responders from non-responders, and correlation analysis shows that high combining predictor was associated with more Treg cells (*P* = 0.009) (supplementary Fig. 4).

**Fig. 2:**
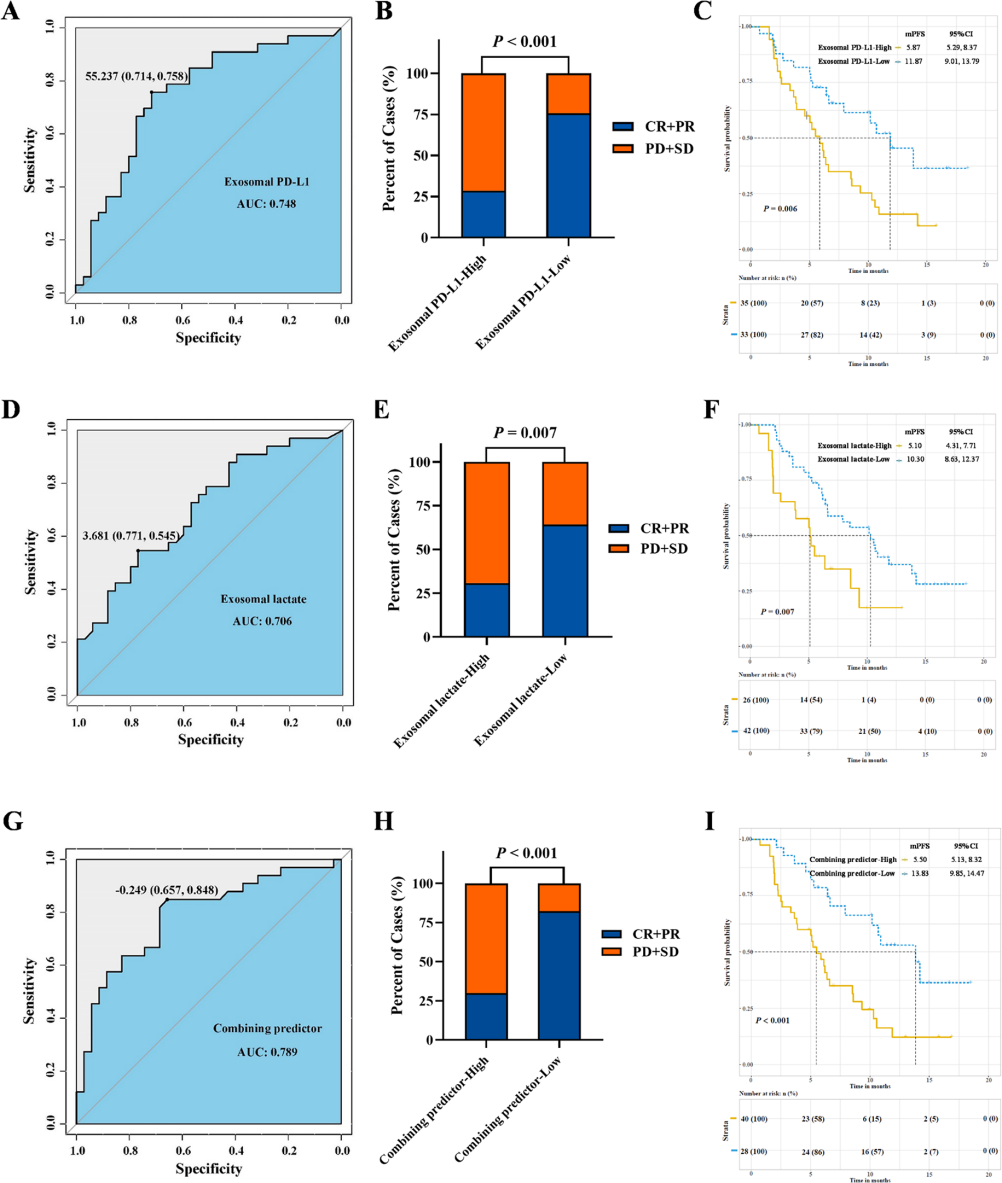
Levels of exosomal PD-L1 and exosomal lactate and treatment efficacy. (**A**, **B**) ROC curve analysis of exosomal PD-L1 levels in responders (CR+PR) and non-responders (SD+PD) (AUC = 0.748, Sensitivity 0.758, *P* < 0.001), low exosomal PD-L1 group showed a better clinical response vs. high exosomal PD-L1 group (ORR: 75.8% vs. 28.6%). (**D**, **E**) ROC curve analysis of exosomal lactate levels in responders and non-responders (AUC = 0.706, Sensitivity 0.545, *P* = 0.004), low lactate PD-L1 group showed a better clinical response vs. high lactate PD-L1 group (ORR: 64.3% vs. 30.8%). (**G**, **H**) ROC curve analysis of PD-L1 and lactate combination levels in exosomes of responders and non-responders (AUC = 0.789, Sensitivity 0.848, *P* < 0.001), Low combining predictor showed a better clinical response vs. high combining predictor group (ORR: 82.1% vs. 30.0%). Chi-square test was used to determine the statistical significance between the groups. Low exosomal PD-L1 level (**C**), low exosomal lactate level (**F**), low level of combining predictor (**I**) was associated with longer progression-free survival

Exosomes were involved in the immune escape and exosomes highly expressing PD-L1 could inhibit antitumor immune responses by inactivating T lymphocytes [6–10]. Our study showed that high exosomal PD-L1 level in plasma was associated with an increase in peripheral blood PD-1^+^ Treg cells in advanced metastatic esophagogastric cancer patients, and compared with PD-1^−^ Treg cells, PD-1^+^ Treg cells proliferate more actively and exhibit strong immunosuppressive effects, resulting in inferior clinical outcomes after first-line PD-1 blockade plus chemotherapy [11]. In tumor immunity, Treg cells can inhibit CD8^+^T cells and the balance between Treg cells and CD8^+^T cells is crucial for achieving superior antitumor efficacy [12–14]. In our study, exosomal lactate level in plasma before treatment was positively correlated with the rate of Treg cells/CD8^+^T cells, and the Treg cell/CD8^+^T cell ratio of responders was significantly lower than that of non-responders, the combination of exosomal PD-L1 and lactate best stratified clinical responders from non-responders. Thus, we speculate that exosomal PD-L1 and lactate have a synergistic effect on the proliferation and activation of Treg cells, exosomal PD-L1 promote the expression of PD-1 in Treg cells, exosomal lactate can provide metabolic support for Treg cells, further promoting immunosuppressive effects and inhibiting antitumor immunity, leading to anti-PD-1 therapy failure.

## Electronic supplementary material

Below is the link to the electronic supplementary material.

Supplementary Material 1

## Data Availability

No datasets were generated or analysed during the current study.
