# Supplementary material for: Exosomal PD-L1 and lactate versus tissue PD-L1 as biomarkers for clinical outcomes of PD-1 Blockade plus chemotherapy in metastatic esophagogastric signet ring cell carcinoma
Source: Exp Hematol Oncol. 2025 Mar 12;14:34. doi: 10.1186/s40164-025-00615-w (PMC11905711; doi:10.1186/s40164-025-00615-w)
Supplement: Supplementary file 1 — Supplementary Material 1 [file 40164_2025_615_MOESM1_ESM.docx]

**CONTENTS:**

**Materials and Methods**

**Supplemental Tables**

Supplementary Table 1. Baseline characteristics of patients.

Supplementary Table 2. Efficacy in different exosomal lactate and exosomal PD-L1 level groups.

**Supplemental Figures**

Supplementary Figure 1. Tumor response and tissue PD-L1, exosomal PD-L1, exosomal lactate.

Supplementary Figure 2. Progression-free survival and exosomal PD-L1 level, exosomal lactate level.

Supplementary Figure 3. Association between tumor response and peripheral blood T cell subsets.

Supplementary Figure 4. Association between the levels of PD-L1 and lactate in exosomes and T cells in peripheral blood.

Supplementary Figure 5. Study schema.

**Materials and Methods**

**Patients and specimen collection**

This was an investigator-initiated, prospective, exploratory study, was approved by the Medical Ethics Committee of Renmin Hospital of Wuhan University (WDRY2023-K093), and was conducted in accordance with the International Ethical Guidelines for Biomedical Research Involving Human Subjects (CIOMS). All study participants were consented in writing for tissue and blood collection. The eligible patients were scheduled to undergo PD-1 blockade (nivolumab 360 mg, every 3 weeks or pembrolizumab 200 mg, every 3 weeks) plus chemotherapy (XELOX [oxaliplatin 130 mg/m^2^, day 1 and capecitabine 1,000 mg/m^2^ twice daily, days 1–14, every 3 weeks], and those receiving at least 2 cycles of treatment were enrolled in this study. Eligible esophagogastric cancer patients were signet ring cell adenocarcinoma, and other major inclusion criteria were as follows: (1) HER-2 negative, (2) initial stage IVB, (3) had measurable lesions for efficacy evaluation, (4) able to provide tumor tissue specimens for PD-L1 expression detection, (5) adequate bone marrow, hepatic, and renal function, (6) baseline Eastern Cooperative Oncology Group (ECOG) performance status of 0-1. Imaging evaluation was performed every 6 weeks until disease progression or unacceptable toxicity occurs, clinical responses were evaluated by Response Evaluation Criteria in Solid Tumors (RECIST) v1.1 criteria.

Peripheral blood plasma was obtained in EDTA containing tubes and the blood samples were delivered to the laboratory and aliquot exactly 200 μL whole blood into the bottom of a tube and vortex for flow cytometry. The rest of whole blood samples were centrifuged at 1,000 g for 10 minutes at 4°C, and then separated into plasma and cellular fractions. The plasma samples were centrifuged at 3,000 g for 15 min at 4°C to remove cell debris and dead cells and then stored in 1 mL aliquots at −80°C until use.

**Immunohistochemistry of LDHA and PD-L1 in** **tumor tissue samples**

Paraffin-embedded tumor slices (4um thickness) were cut and transferred onto a glass slide, dehydrated in environmentally friendly dewaxing transparent liquid (G1128, Servicebio) and a series of alcohol concentrations (100%, 95%, 85%, 70%). Subsequently, the slides were in ethylene diamine tetra acetic acid (EDTA, pH9.0) buffer and subjected to microwave oven irradiation for antigen retrieval. After blocked with 3% bovine serum albumin (BSA) for 30 min, the slides were incubated with the primary antibody (Abcam, Ab52488, dilution 1:1000) at 4ºC overnight. After washing in PBS for three times, the slides were incubated with HRP-conjugated anti-rabbit IgG (GB23303, Servicebio) at room temperature for 50 min and stained with DAB kit (G1212, Servicebio), and counterstained using hematoxylin (G1004, Servicebio). After staining, the slides were digitally scanned using the Pannoramic 250 FLASH scanner (3DHISTECH, Hungary), and analyzed with Pannoramic Viewer1software (3DHISTECH, Hungary). PD-L1 expression was determined by immunohistochemistry (22C3 pharmDx assay, Agilent, Dako). Combined positive score (CPS) was calculated by adding the number of viable PD-L1 positive tumor cells to the number of positive tumor-infiltrating immune cells, divided by the total number of viable tumor cells, with a maximum score of 100. We set a range of thresholds for CPS (≥1 or 10).

**Isolation of exosomes from peripheral blood plasma**

2 mL plasma specimens were thawed immediately prior exosome isolation and were differentially centrifuged 3,000 g for 10 min at 4°C and then 12,000 g for 30 min at 4°C. Next, supernatants obtained in the previous step were ultra-centrifuged at 100,000 g for 90 min at 4°C (Beckman Coulter, Optima XE-100) to pellet the exosomes. Then the pelleted exosomes were washed with phosphate buffer solution (PBS) and ultra-centrifuged again at 100,000 g for 90 min at 4°C. The exosomes were resuspended in PBS and stored at -80°C until use. A bicinchoninic acid (BCA) assay kit (P0010S, Beyotime, China) was used to quantify the concentration of exosomes.

**Transmission electron microscope (TEM) of exosomes**

About 20 uL of the exosomes samples were added dropwise to 200-mesh grids and incubated at room temperature for 10 min, then the grids were negatively stained with 2% phosphotungsticacid for 3 min, and the remaining liquid was removed by filter paper. Then observed with a HT7800 transmission electron microscope.

**Nanoparticle tracking analysis (NTA) of exosomes**

The size distribution and concentration of isolated exosomes were measured using Zeta View_-_Particle Metrix (Particle Metrix, PMX-120, Germany). The data were analyzed using NTA software.

**Western blot analysis**

Exosomal proteins were denatured with a loading buffer and were separated using 10% sodium dodecyl sulfate–polyacrylamide gel electrophoresis (SDS-PAGE) and transferred onto polyvinylidene fluoride (PVDF) membranes. The blots were blocked with 5% bovine serum albumin for 1 h at room temperature, and incubated overnight at 4 ºC with antibodies specific for CD9 (ab263019, Abcam), CD63 (ab134045, Abcam), CD81 (ab109201, Abcam), TSG101 (ab125011, Abcam), Hsp70 (ab181606, Abcam), PD-L1 (29074-1, Signalway Antibody), followed by incubation with HRP-conjugated anti-rabbit IgG (GB23303, Servicebio) at room temperature for 1 h. The blots on the membranes were revealed by using ECL detection reagents (G2014, Servicebio).

**ELISA**

PD-L1 in exosomes levels were measured using an enzyme-linked immunosorbent assay (PD-L1 Human ELISA Kit, Abcam, ab277712), following vendor’s protocol. Lysates in exosomes were prepared in RIPA buffer supplemented with Halt Protease and Phosphatase Inhibitor. Lactate levels were determined enzymatically using a lactate assay kit II (Abcam, ab65330) according to the manufacturer’s instructions.

**Flow cytometry of patients’ peripheral blood sample**

Aliquot exactly 200 μL whole blood into the bottom of a tube and vortex, then add 2 mL of diluted 1× Lysing Buffer (BD, 555899) and vortex immediately, incubate at RT for 15 min in the dark to lyse red blood cells. The samples were centrifuged at 300 g for 5 min at RT to pellet the white blood cells, then the pelleted white blood cells were washed with PBS and centrifuged at 300 g for 5 min at RT. The white blood cells were resuspended in 100 ul PBS, and subjected to staining with master mix of antibodies for surface stains including CD3 (BD, 563109), CD4 (BD, 566392), CD8 (BD, 557834), CD25 (BD, 564467), CD127 (BD, 558598), CD279 (BD, 562516) for 15 min at RT in the dark. After washing, cells were resuspended in 200 μL PBS for data acquisition on a Beckman Coulter CytoFlex and analyzed with and Flow Jo software. The experiment protocol, the representative TEM image and the size distribution and concentration of exosomes were shown in Supplementary Fig. 5.

**Statistical analysis**

For descriptive statistics, continuous variables with approximate normal distributions were expressed as mean (±standard deviation), while for continuous variables with asymmetrical distributions, they were expressed as median and interquartile range. Categorical variables were expressed by percentage. We used chi-square test to compare categorical variables, and for continuous data, Student's t-test or the Mann–Whitney U test were performed. To evaluate the predicted value of exosomal PD-L1 and lactate for responders, the receiver operating characteristic (ROC) curves were created, and the area under the curve (AUC) summarizes the performance of exosomal PD-L1 and lactate. The cutoff values were the continuous variables with the maximum sum of sensitivity and specificity in detection. Correlational analyses were performed using the Pearson correlation test, rates of PFS was estimated by the Kaplan-Meier method, and the log-rank test was used to compare the differences between groups.

Supplementary Table 1. Baseline characteristics of patients (No. [%])

| **Characteristic** | **All patients**  (**N**=**68**) | **Responders (CR+PR)**  **(N=35)** | **Non-responders (SD+PD)**  **(N=33)** | ***P*** |
| --- | --- | --- | --- | --- |
| **Sex,**  **Male/Female** | 46/22  (67.6%/32.4%) | 26/9  (74.3%/25.7%) | 20/13  (60.6%/39.4%) | 0.228 |
| **Age, years**  **＜65 /≥65** | 35/33  (51.5%/48.5%) | 16/19  (45.7%/54.3%) | 19/14  (57.6%/42.4%) | 0.328 |
| **Primary tumor site Gastric/GEJ** | 45/23  （66.2%/33.8%） | 23/12  （65.7%/34.3%） | 22/11  （66.7%/33.3%） | 0.934 |
| ***p53*, mutant/wild type** | 14/18  （43.8%/56.3%） | 7/10  （41.2%/58.8%） | 7/8  （46.7%/53.3%） | 0.755 |
| **Ki-67 labeling index** | 0.67 ±0.17 | 0.68 ± 0.14 | 0.66 ± 0.20 | 0.702 |
| **CA50 (U/mL)** | 61.39 ± 159.04 | 54.22 ± 167.61 | 68.56 ± 152.97 | 0.749 |
| **CA199 (U/mL)** | 10.34 (4.03, 26.67) | 9.37 (2.93, 23.19) | 11.92 (5.28, 29.40) | 0.398 |
| **CEA (ng/mL)** | 2.50 (1.21, 6.64) | 1.85 (1.05, 4.04) | 3.25 (1.52, 11.57) | 0.061 |
| **LDHA (U/L)** | 216.00 (171.00, 261.00) | 213.00 (167.00, 241.00) | 220.50 (184.25, 355.00) | 0.269 |

Note: Continuous variables with approximate normal distributions were expressed as mean (±standard deviation), while continuous variables with asymmetrical distributions were expressed as median and interquartile range. Categorical variables were expressed by percentage. Abbreviation: GEJ, gastroesophageal junction, CEA = carcinoma embryonic antigen, CA19-9 = carbohydrate antigen 19-9, CA50 = carbohydrate antigen 50, LDHA = lactate dehydrogenase A.

Supplementary Table 2. Efficacy in different exosomal lactate and exosomal PD-L1 level groups (No. [%])

| **Best response** | **All patients（N=68）** | **Exosomal lactate** | | ***P*** | **Exosomal PD-L1** | | ***P*** | **Combination of exosomal PD-L1 and lactate** | | ***P*** |
| --- | --- | --- | --- | --- | --- | --- | --- | --- | --- | --- |
|  |  | **High(N=26)** | **Low(N=42)** |  | **High(N=35)** | **Low(N=33)** |  | **High(N=40)** | **Low(N=28)** |  |
| **CR** | 3 (4.4%) | 0 (0%) | 3(7.1%) | 0.163 | 0(0%) | 3(9.1%) | 0.068 | 0(0%) | 3(10.7%) | 0.034 |
| **PR** | 32 (47.1%) | 8 (30.8%) | 24(57.1%) | 0.034 | 10(28.6%) | 22(66.7%) | 0.002 | 12(30.0%) | 20(71.4%) | 0.001 |
| **SD** | 24 (35.3%) | 12(46.2%) | 12(28.6%) | 0.140 | 20(57.1%) | 4(12.1%) | <0.001 | 21(52.5%) | 3(10.7%) | <0.001 |
| **PD** | 9 (13.2%) | 6(23.1%) | 3(7.1%) | 0.060 | 5(14.3%) | 4(12.1%) | 0.792 | 7(17.5%) | 2(7.1%) | 0.215 |
| **ORR** | 35 (51.5%) | 8(30.8%) | 27(64.3%) | 0.007 | 10(28.6%) | 25(75.8%) | <0.001 | 12(30.0%) | 23(82.1%) | <0.001 |
| **mPFS (months)** | 6.63 | 5.10 | 10.30 | 0.007 | 5.87 | 11.87 | 0.006 | 5.50 | 13.83 | <0.001 |

Abbreviation: CR = complete response; PR = partial response; SD = stable disease; PD = progressive disease; ORR = objective response rate; mPFS = median progression-free survival.


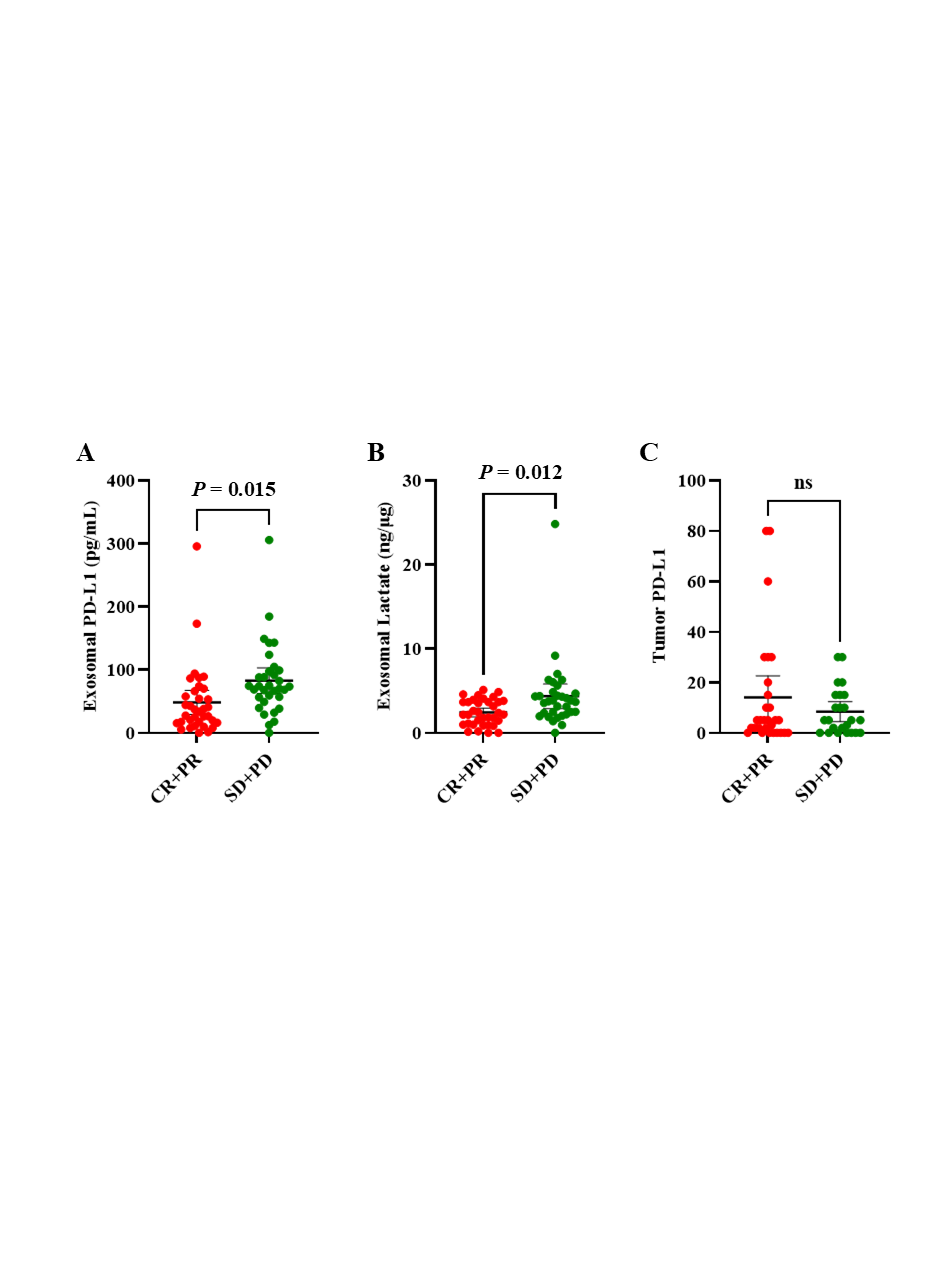


Supplementary Figure 1. Tumor response and tissue PD-L1, exosomal PD-L1, exosomal lactate. Relationship between tumor response and levels of plasma exosomal PD-L1 (A), levels of plasma exosomal lactate (B), tumor tissue PD-L1 expression levels (C).


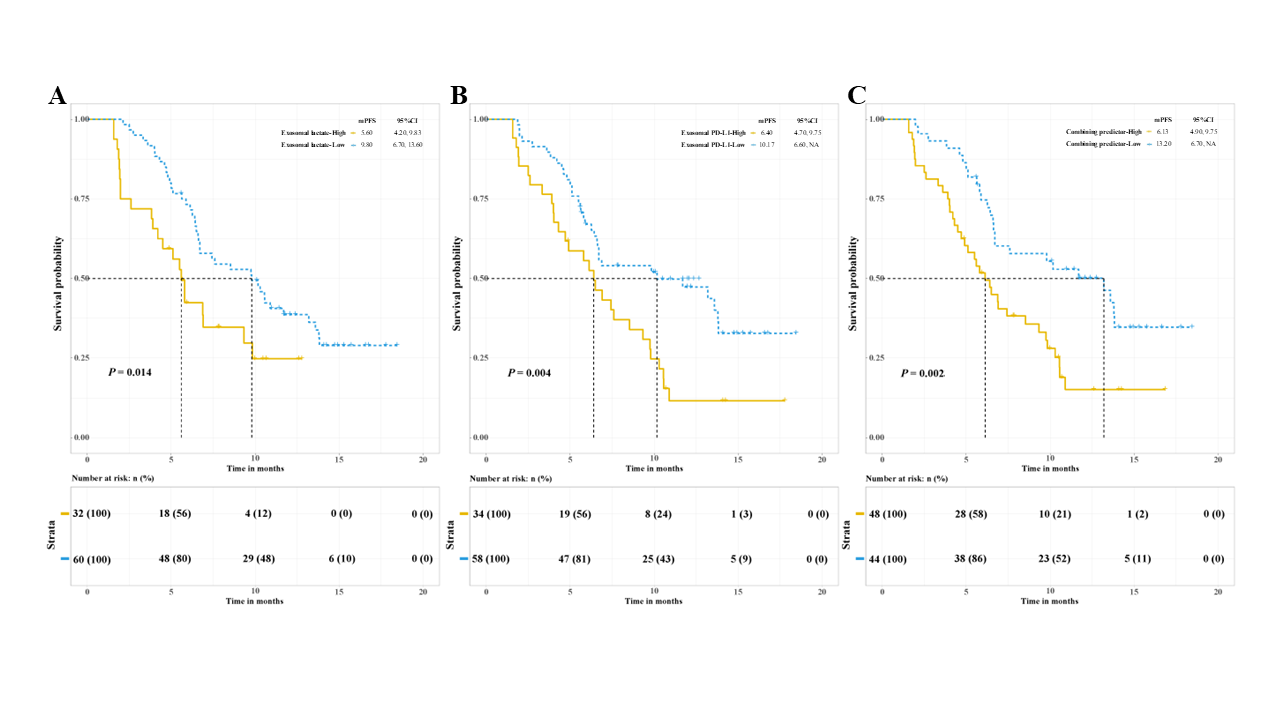


Supplementary Figure 2. Progression-free survival (PFS) and exosomal PD-L1 level, exosomal lactate level. 92 HER2–negative patients with metastatic esophagogastric signet ring cell carcinoma were used for the external validation. According to the optimal critical value determined by the ROC curve, compared to those with high level of exosomal lactate or exosomal PD-L1, patients presented with exosomal lactate <3.681 ng/ug (A), exosomal PD-L1 <55.237 pg/ml (B) or the combining predictor of exosomal lactate and PD-L1 lower than -0.249 (C) before treatment achieved a prolonged median PFS.


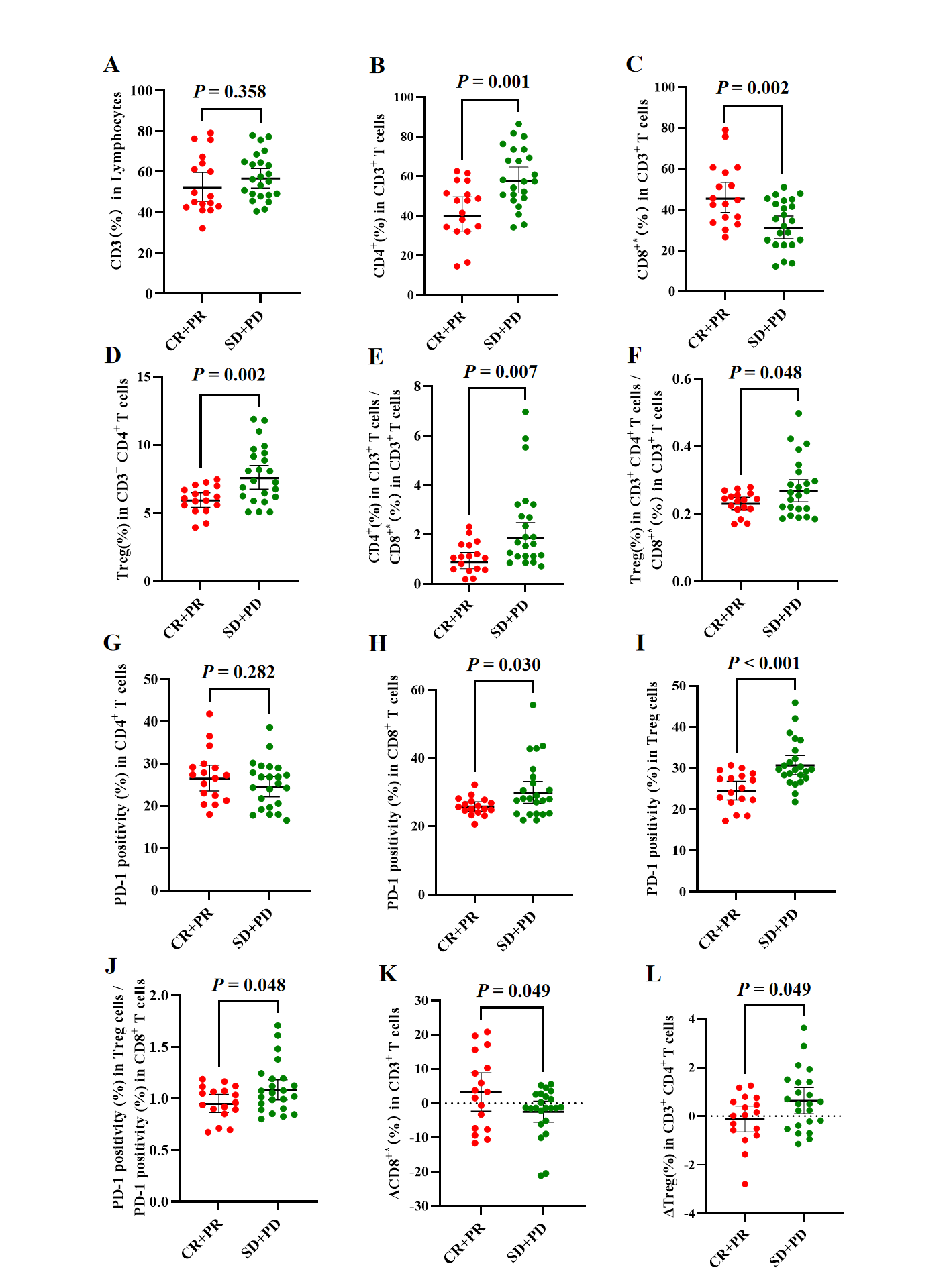


Supplementary Figure 3. Association between tumor response and peripheral blood T cell subsets. Comparison of ratio of CD3^+^ T cells (A), CD4^+^ T cells (B), CD8^+^ T cells (C), Treg cells (D), CD4^+^ T cells/CD8^+^ T cells (E), Treg cells/CD8^+^ T cells (F) in responders (CR+PR) and non-responders (SD+PD). Comparison of ratio of PD-1 positivity (%) in CD4^+^ T cells (G), CD8^+^ T cells (H), Treg cells (I) and PD-1 positivity (%) in Treg cells/PD-1 positivity (%) in CD8^+^ T cells (J). Changes in CD8^+^T cells ratio (K) and Treg cells ratio (L) after treatment in responders and non-responders.


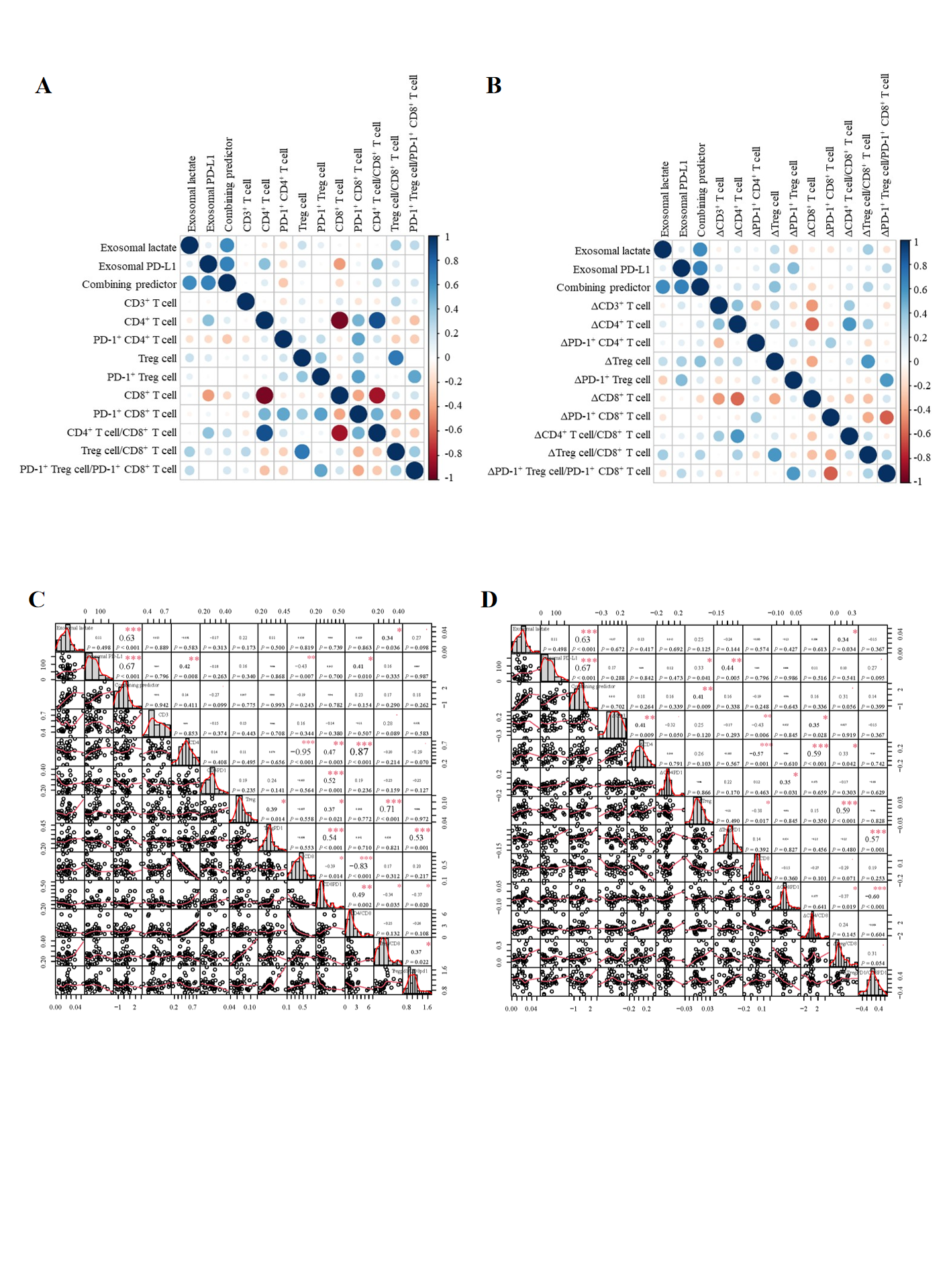


Supplementary Figure 4. Association between the levels of PD-L1 and lactate in exosomes and T cells in peripheral blood. Pearson correlation between the levels of PD-L1 and lactate in exosomes and T cells in peripheral blood before treatment (A, C). Pearson correlation between the levels of PD-L1 and lactate in exosomes and changes in peripheral blood T cells after treatment (B, D) .


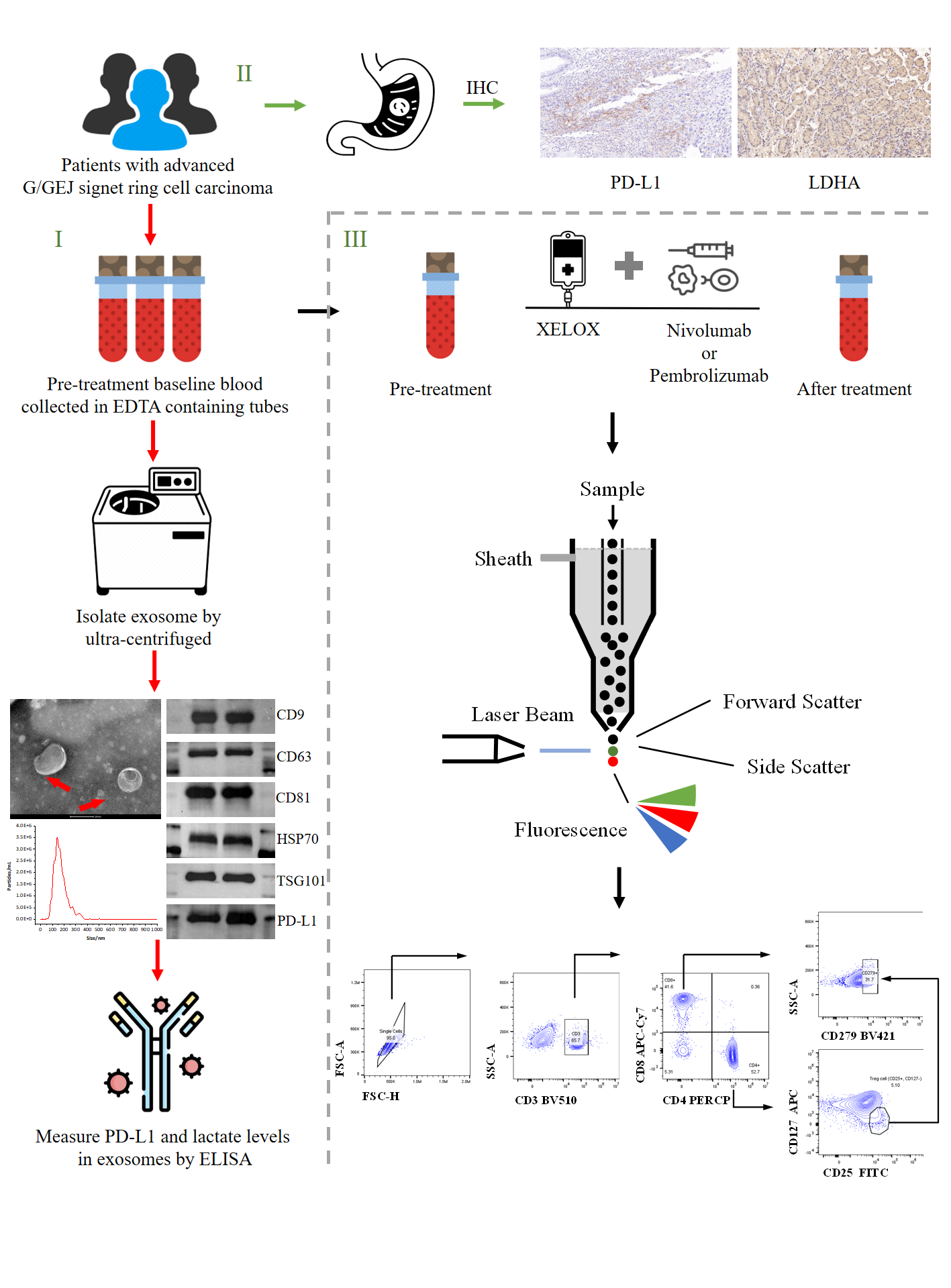


Supplementary Figure 5. Study schema. Plasma exosomes were isolated by differential centrifugation before treatment initiation and verified using TEM, NTA analysis and Western blot. Exosomal PD-L1 concentrations were measured using a PD-L1 Human ELISA Kit, exosomal lactate concentrations were determined enzymatically using a lactate assay kit, and the levels of PD-L1 and lactate were calculated according to standard curves (I). Immunohistochemical staining for PD-L1 and LDHA were performed on tumor tissue samples (II). Blood samples of pre-treatment baseline and after treatment were collected in EDTA containing tubes, the composition of peripheral CD3^+^ T cells, CD4^+^Tcells, CD8^+^T cells, regulatory T (CD4^+^CD25^+^CD127^low^, Treg) cells, and the expression of their PD-1 were assessed by flow cytometry (III).
